# DeePromoter: Robust Promoter Predictor Using Deep Learning

**DOI:** 10.3389/fgene.2019.00286

**Published:** 2019-04-05

**Authors:** Mhaned Oubounyt, Zakaria Louadi, Hilal Tayara, Kil To Chong

**Affiliations:** ^1^Department of Information and Electronics Engineering, Chonbuk National University, Jeonju, South Korea; ^2^Advanced Research Center of Information and Electronics Engineering, Chonbuk National University, Jeonju, South Korea

**Keywords:** promoter, DeePromoter, bioinformatics, deep learning, convolutional neural network

## Abstract

The promoter region is located near the transcription start sites and regulates transcription initiation of the gene by controlling the binding of RNA polymerase. Thus, promoter region recognition is an important area of interest in the field of bioinformatics. Numerous tools for promoter prediction were proposed. However, the reliability of these tools still needs to be improved. In this work, we propose a robust deep learning model, called DeePromoter, to analyze the characteristics of the short eukaryotic promoter sequences, and accurately recognize the human and mouse promoter sequences. DeePromoter combines a convolutional neural network (CNN) and a long short-term memory (LSTM). Additionally, instead of using non-promoter regions of the genome as a negative set, we derive a more challenging negative set from the promoter sequences. The proposed negative set reconstruction method improves the discrimination ability and significantly reduces the number of false positive predictions. Consequently, DeePromoter outperforms the previously proposed promoter prediction tools. In addition, a web-server for promoter prediction is developed based on the proposed methods and made available at https://home.jbnu.ac.kr/NSCL/deepromoter.htm.

## 1. Introduction

Promoters are the key elements that belong to non-coding regions in the genome. They largely control the activation or repression of the genes. They are located near and upstream the gene's transcription start site (TSS). A gene's promoter flanking region may contain many crucial short DNA elements and motifs (5 and 15 bases long) that serve as recognition sites for the proteins that provide proper initiation and regulation of transcription of the downstream gene (Juven-Gershon et al., 2008). The initiation of gene transcript is the most fundamental step in the regulation of gene expression. Promoter core is a minimal stretch of DNA sequence that conations TSS and sufficient to directly initiate the transcription. The length of core promoter typically ranges between 60 and 120 base pairs (bp).

The TATA-box is a promoter subsequence that indicates to other molecules where transcription begins. It was named “TATA-box” as its sequence is characterized by repeating T and A base pairs (TATAAA) (Baker et al., 2003). The vast majority of studies on the TATA-box have been conducted on human, yeast, and Drosophila genomes, however, similar elements have been found in other species such as archaea and ancient eukaryotes (Smale and Kadonaga, 2003). In human case, 24% of genes have promoter regions containing TATA-box (Yang et al., 2007). In eukaryotes, TATA-box is located at ~25 bp upstream of the TSS (Xu et al., 2016). It is able to define the direction of transcription and also indicates the DNA strand to be read. Proteins called transcription factors bind to several non-coding regions including TATA-box and recruit an enzyme called RNA polymerase, which synthesizes RNA from DNA.

Due to the important role of the promoters in gene transcription, accurate prediction of promoter sites become a required step in gene expression, patterns interpretation, and building and understanding the functionality of genetic regulatory networks. There were different biological experiments for identification of promoters such as mutational analysis (Matsumine et al., 1998) and immunoprecipitation assays (Kim et al., 2004; Dahl and Collas, 2008). However, these methods were both expensive and time-consuming. Recently, with the development of the next-generation sequencing (NGS) (Behjati and Tarpey, 2013) more genes of different organisms have been sequenced and their gene elements have been computationally explored (Zhang et al., 2011). On the other hand, the innovation of NGS technology has resulted in a dramatic fall of the cost of the whole genome sequencing, thus, more sequencing data is available. The data availability attracts researchers to develop computational models for promoter prediction task. However, it is still an incomplete task and there is no efficient software that can accurately predict promoters.

Promoter predictors can be categorized based on the utilized approach into three groups namely signal-based approach, content-based approach, and the GpG-based approach. Signal-based predictors focus on promoter elements related to RNA polymerase binding site and ignore the non-element portions of the sequence. As a result, the prediction accuracy was weak and not satisfying. Examples of signal-based predictors include: PromoterScan (Prestridge, 1995) which used the extracted features of the TATA-box and a weighted matrix of transcription factor binding sites with a linear discriminator to classify promoter sequences form non-promoter ones; Promoter2.0 (Knudsen, 1999) which extracted the features from different boxes such as TATA-Box, CAAT-Box, and GC-Box and passed them to artificial neural networks (ANN) for classification; NNPP2.1 (Reese, 2001) which utilized initiator element (Inr) and TATA-Box for feature extraction and a time-delay neural network for classification, and Down and Hubbard (2002) which used TATA-Box and utilized a relevance vector machines (RVM) as a classifier. Content-based predictors relied on counting the frequency of k-mer by running a k-length window across the sequence. However, these methods ignore the spatial information of the base pairs in the sequences. Examples of Content-based predictors include: PromFind (Hutchinson, 1996) which used the k-mer frequency to perform the hexamer promoter prediction; PromoterInspector (Scherf et al., 2000) which identified the regions containing promoters based on a common genomic context of polymerase II promoters by scanning for specific features defined as variable length motifs; MCPromoter1.1 (Ohler et al., 1999) which used a single interpolated Markov chain (IMC) of 5th order to predict promoter sequences. Finally, GpG-based predictors utilized the location of GpG islands as the promoter region or the first exon region in the human genes usually contains GpG islands (Ioshikhes and Zhang, 2000; Davuluri et al., 2001; Lander et al., 2001; Ponger and Mouchiroud, 2002). However, only 60% of the promoters contain GpG islands, therefore the prediction accuracy of this kind of predictors never exceeded 60%.

Recently, sequence-based approaches have been utilized for promoter prediction. Yang et al. (2017) utilized different feature extraction strategies to capture the most relevant sequence information in order to predict enhancer-promoter interactions. Lin et al. (2017) proposed a sequence-based predictor, named “iPro70-PseZNC”, for sigma70 promoter's identification in the prokaryote. Likewise, Bharanikumar et al. (2018) proposed PromoterPredict in order to predict the strength of *Escherichia coli* promoters based on a dynamic multiple regression approach where the sequences were represented as position weight matrices (PWM). Kanhere and Bansal (2005) utilized the differences in DNA sequence stability between the promoter and non-promoter sequences in order to distinguish them. Xiao et al. (2018) introduced a two layers predictor called iPSW(2L)-PseKNC for promoter sequences identification as well as the strength of the promoters by extracting hybrid features from the sequences.

All of the aforementioned predictors require domain-knowledge in order to hand-craft the features. On the other hand, deep learning based approaches enable building more efficient models using raw data (DNA/RNA sequences) directly. Deep convolutional neural network achieved state-of–the-art results in challenging tasks such as processing image, video, audio, and speech (Krizhevsky et al., 2012; LeCun et al., 2015; Schmidhuber, 2015; Szegedy et al., 2015). In addition, it was successfully applied in biological problems such as DeepBind (Alipanahi et al., 2015), DeepCpG (Angermueller et al., 2017), branch point selection (Nazari et al., 2018), alternative splicing sites prediction (Oubounyt et al., 2018), 2'-Omethylation sites prediction (Tahir et al., 2018), DNA sequence quantification (Quang and Xie, 2016), human protein subcellular localization (Wei et al., 2018), etc. Furthermore, CNN recently gained significant attention in the promoter recognition task. Very recently, Umarov and Solovyev (2017) introduced CNNprom for short promoter sequences discrimination, this CNN based architecture achieved high results in classifying promoter and non-promoter sequences. Afterward, this model was improved by Qian et al. (2018) where the authors used support vector machine (SVM) classifier to inspect the most important promoter sequence elements. Next, the most influential elements were kept uncompressed while compressing the less important ones. This process resulted in better performance. Recently, long promoter identification model was proposed by Umarov et al. (2019) in which the authors focused on the identification of TSS position.

In all the above-mentioned works the negative set was extracted from non-promoter regions of the genome. Knowing that the promoter sequences are rich exclusively of specific functional elements such as TATA-box which is located at –30~–25 bp, GC-Box which is located at –110~–80 bp, CAAT-Box which is located at –80~–70 bp, etc. This results in high classification accuracy in due to huge disparity between the positive and negative samples in terms of sequence structure. Additionally, the classification task becomes effortless to achieve, for instance, the CNN models will just rely on the presence or absence of some motifs at their specific positions to make the decision on the sequence type. Thus, these models have very low precision/sensitivity (high false positive) when they are tested on genomic sequences that have promoter motifs but they are not promoter sequences. It is well known that there are more TATAAA motifs in the genome than the ones belonging to the promoter regions. For instance, alone the DNA sequence of the human chromosome 1, ftp://ftp.ensembl.org/pub/release-57/fasta/homo_sapiens/dna/, contains 151 656 TATAAA motifs. It is more than the approximated maximal number of genes in the total human genome. As an illustration of this issue, we notice that when testing these models on non-promoter sequences that have TATA-box they misclassify most of these sequences. Therefore, in order to generate a robust classifier, the negative set should be selected carefully as it determines the features that will be used by the classifier in order to discriminate the classes. The importance of this idea has been demonstrated in previous works such as (Wei et al., 2014). In this work, we mainly address this issue and propose an approach that integrates some of the positive class functional motifs in the negative class to reduce the model's dependency on these motifs. We utilize a CNN combined with LSTM model to analyze sequence characteristics of human and mouse TATA and non-TATA eukaryotic promoters and build computational models that can accurately discriminate short promoter sequences from non-promoter ones.

## 2. Materials and Methods

### 2.1. Dataset

The datasets, which are used for training and testing the proposed promoter predictor, are collected from human and mouse. They contain two distinctive classes of the promoters namely TATA promoters (i.e., the sequences that contain TATA-box) and non-TATA promoters. These datasets were built from Eukaryotic Promoter Database (EPDnew) (Dreos et al., 2012). The EPDnew is a new section under the well-known EPD dataset (Périer et al., 2000) which is annotated a non-redundant collection of eukaryotic POL II promoters where transcription start site has been determined experimentally. It provides high-quality promoters compared to ENSEMBL promoter collection (Dreos et al., 2012) and it is publically accessible at https://epd.epfl.ch//index.php. We downloaded TATA and non-TATA promoter genomic sequences for each organism from EPDnew. This operation resulted in obtaining four promoter datasets namely: Human-TATA, Human-non-TATA, Mouse-TATA, and Mouse-non-TATA. For each of these datasets, a negative set (non-promoter sequences) with the same size of the positive one is constructed based on the proposed approach as described in the following section. The details on the numbers of promoter sequences for each organism are given in Table 1. All sequences have a length of 300 bp and were extracted from -249~+50 bp (+1 refers to TSS position). As a quality control, we used 5-fold cross-validation to assess the proposed model. In this case, 3-folds are used for training, 1-fold is used for validation, and the remaining fold is used for testing. Thus, the proposed model is trained 5 times and the overall performance of the 5-fold is calculated.

**Table 1:** Statistics of the four datasets used in this study.

**Oganism**	**Promoter seq**.	**Non promoter seq**.	**Length (bp)**	**Location**
Human-TATA	3,065	3,065	300	–249~50
Human-non-TATA	26,532	26,532	300	–249~50
Mouse-TATA	3,305	3,305	300	–249~50
Mouse-non-TATA	21,804	21,804	300	–249~50

### 2.2. Negative Dataset Construction

In order to train a model that can accurately perform promoter and non-promoter sequences classification, we need to choose the negative set (non-promoter sequences) carefully. This point is crucial in making a model capable of generalizing well, and therefore able to maintain its precision when evaluated on more challenging datasets. Previous works, such as (Qian et al., 2018), constructed negative set by randomly selecting fragments from genome non-promoter regions. Obviously, this approach is not completely reasonable because if there is no intersection between positive and negative sets. Thus, the model will easily find basic features to separate the two classes. For instance, TATA motif can be found in all positive sequences at a specific position (normally 28 bp upstream of the TSS, between –30 and –25 pb in our dataset). Therefore, creating negative set randomly that does not contain this motif will produce high performance in this dataset. However, the model fails at classifying negative sequences that have TATA motif as promoters. In brief, the major flaw in this approach is that when training a deep learning model it only learns to discriminate the positive and negative classes based the presence or absence of some simple features at specific positions, which makes these models impracticable. In this work, we aim to solve this issue by establishing an alternative method to derive the negative set from the positive one.

**Figure 1:**
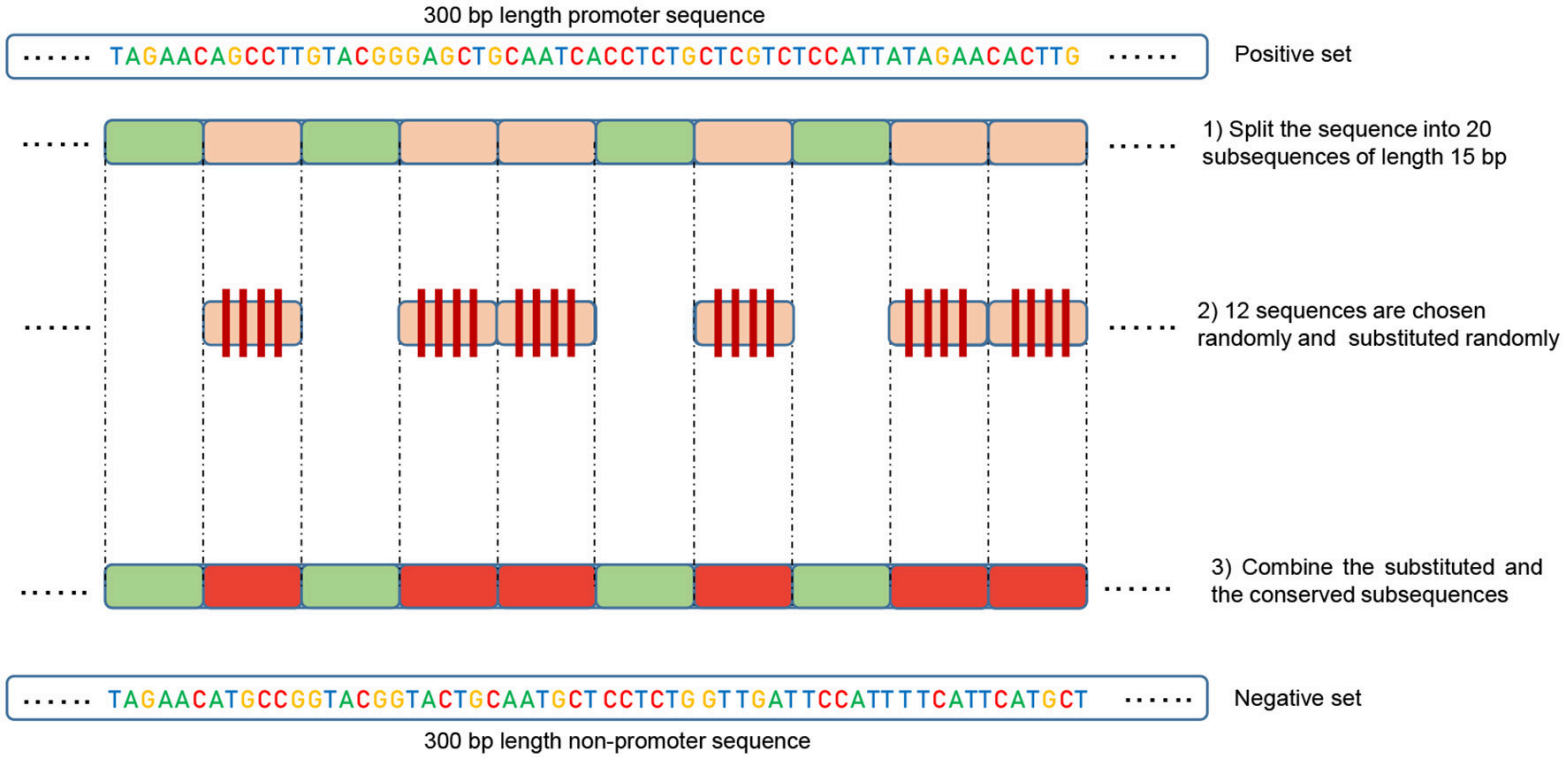
Illustration of the negative set construction method. Green represents the randomly conserved subsequences while red represents the randomly chosen and substituted ones.

**Figure 2:**
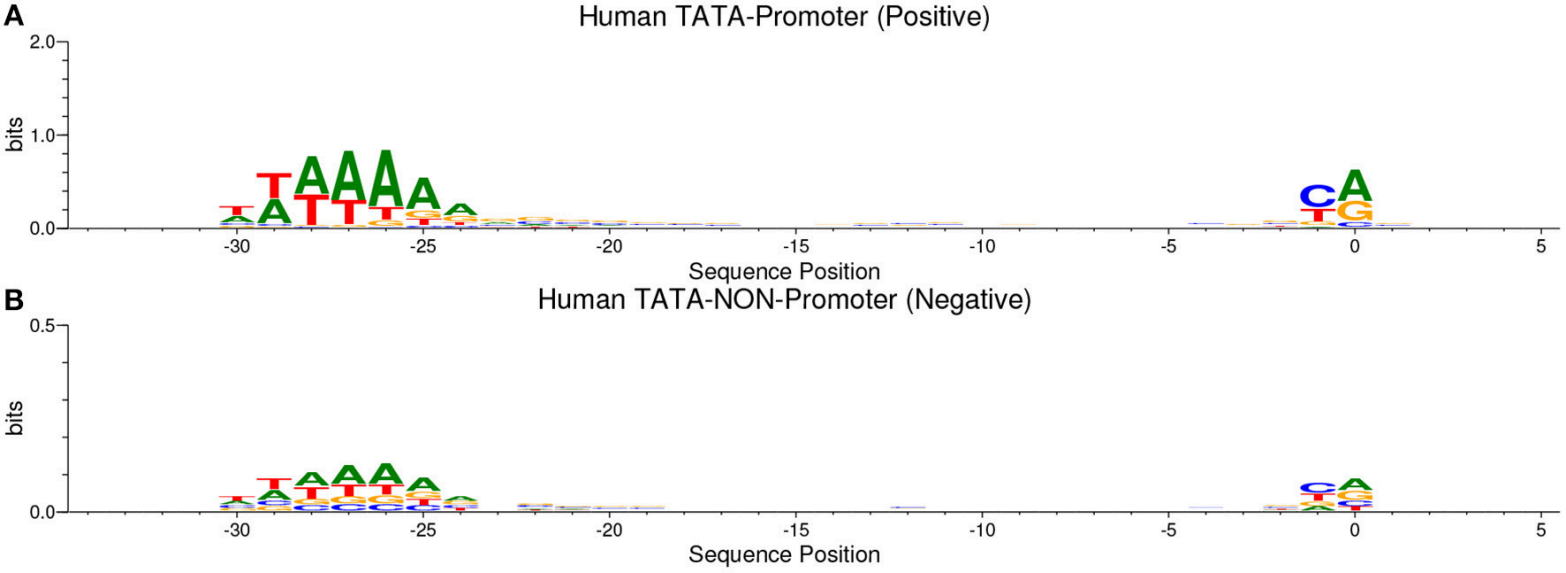
The sequence logo in human TATA promoter for both positive set **(A)** and negative set **(B)**. The plots show the conservation of the functional motifs between the two sets.

**Figure 3:**
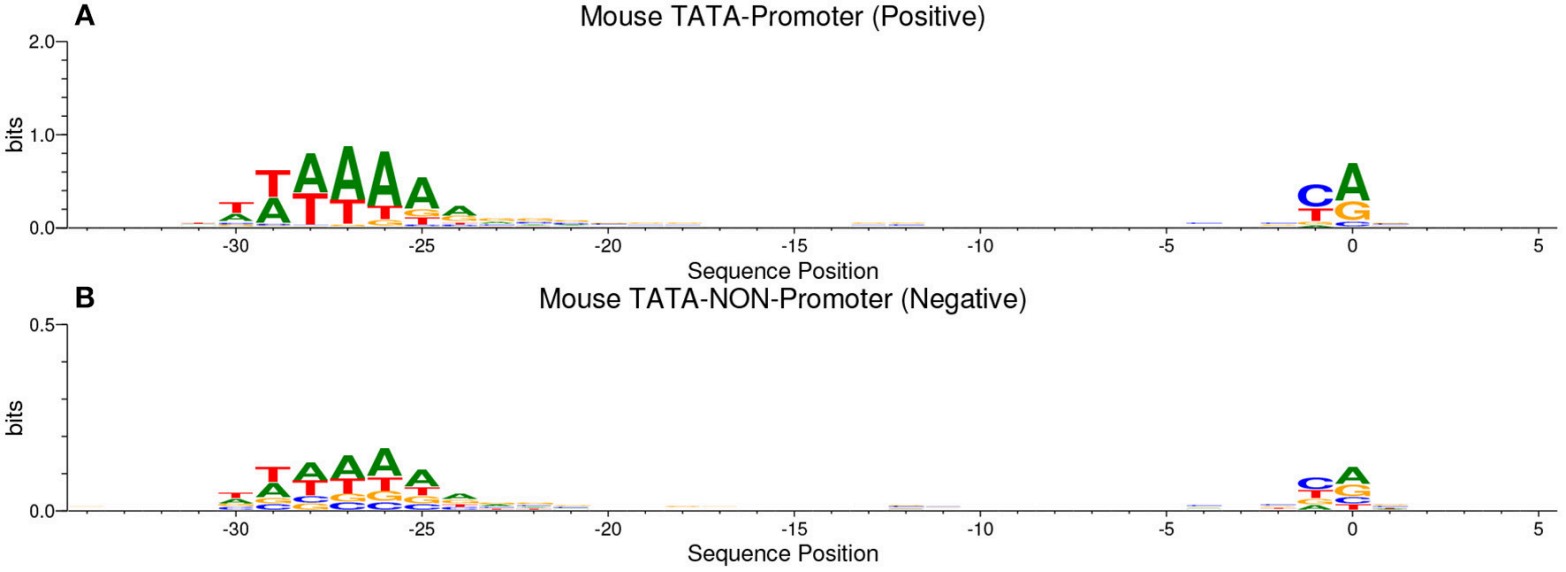
The sequence logo in mouse TATA promoter for both positive set **(A)** and negative set **(B)**. The plots show the conservation of the functional motifs between the two sets.

Our method is based on the fact that whenever the features are common between the negative and the positive class the model tends, when making the decision, to ignore or reduce its dependency on these features (i.e., assign low weights to these features). Instead, the model is forced to search for deeper and less obvious features. Deep learning models generally suffer from slow convergence while training on this type of data. However, this method improves the robustness of the model and ensures generalization. We reconstruct the negative set as follows. Each positive sequence generates one negative sequence. The positive sequence is divided into 20 subsequences. Then, 12 subsequences are picked randomly and substituted randomly. The remaining 8 subsequences are conserved. This process is illustrated in Figure 1. Applying this process to the positive set results in new non-promoter sequences with conserved parts from promoter sequences (the unchanged subsequences, 8 subsequences out of 20). These parameters enable generating a negative set that has 32 and 40% of its sequences containing conserved portions of promoter sequences. This ratio is found to be optimal for having robust promoter predictor as explained in section 3.2. Because the conserved parts occupy the same positions in the negative sequences, the obvious motifs such as TATA-box and TSS are now common between the two sets with a ratio of 32~40%. The sequence logos of the positive and negative sets for both human and mouse TATA promoter data are shown in Figures 2, 3, respectively. It can be seen that the positive and the negative sets share the same basic motifs at the same positions such as TATA motif at the position -30 and –25 bp and the TSS at the position +1 bp. Therefore, the training is more challenging but the resulted model generalizes well.

### 2.3. The Proposed Models

**Figure 4:**
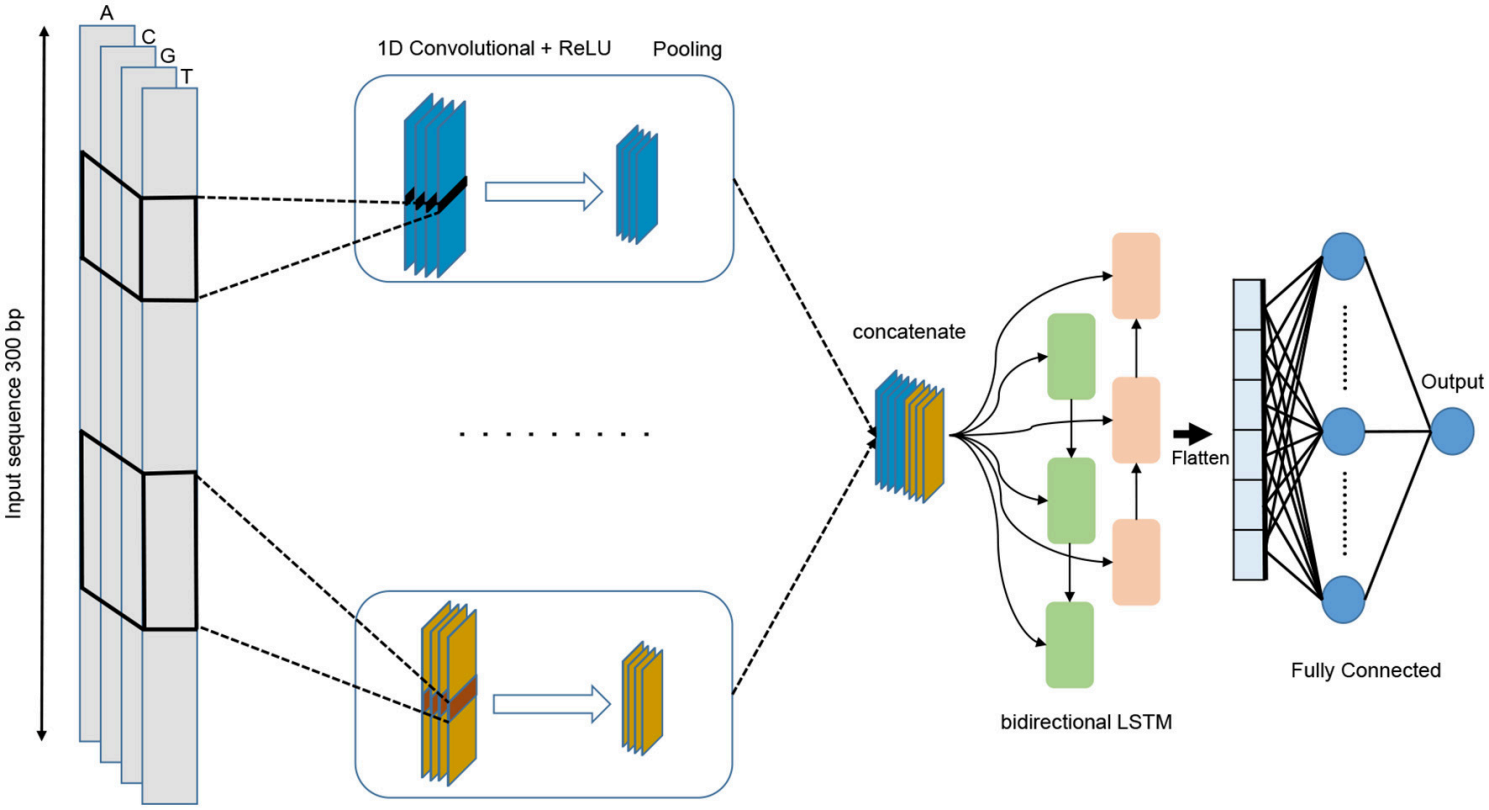
The architecture of the proposed DeePromoter model.

We propose a deep learning model that combines convolution layers with recurrent layers as shown in Figure 4. It accepts a single raw genomic sequence, S={*N*_1_, *N*_2_, …, *N*_*l*_} where N∈ {A, C, G, T} and *l* is the length of the input sequence, as input and outputs a real-valued score. The input is one-hot encoded and represented as a one-dimensional vector with four channels. The length of the vector *l*=300 and the four channels are A, C, G, and T and represented as (1 0 0 0), (0 1 0 0), (0 0 1 0), (0 0 0 1), respectively. In order to select the best performing model, we have used grid search method for choosing the best hyper-parameters. We have tried different architectures such as CNN alone, LSTM alone, BiLSTM alone, CNN combined with LSTM. The tuned hyper-parameters are the number of convolution layers, kernel size, number of filters in each layer, the size of the max pooling layer, dropout probability, and the units of Bi-LSTM layer.

The proposed model starts with multiple convolution layers that are aligned in parallel and help in learning the important motifs of the input sequences with different window size. We use three convolution layers for non-TATA promoter with window sizes of 27, 14, and 7, and two convolution layers for TATA promoters with window sizes of 27, 14. All convolution layers are followed by ReLU activation function (Glorot et al., 2011), a max pooling layer with a window size of 6, and a dropout layer of a probability 0.5. Then, the outputs of these layers are concatenated together and fed into a bidirectional long short-term memory (BiLSTM) (Schuster and Paliwal, 1997) layer with 32 nodes in order to capture the dependencies between the learnt motifs from the convolution layers. The learnt features after BiLSTM are flattened and followed by dropout with a probability of 0.5. Then we add two fully connected layers for classification. The first one has 128 nodes and followed by ReLU and dropout with a probability of 0.5 while the second layer is used for prediction with one node and sigmoid activation function. BiLSTM allows the information to persist and learn long-term dependencies of sequential samples such as DNA and RNA. This is achieved through the LSTM structure which is composed of a memory cell and three gates called input, output, and forget gates. These gates are responsible for regulating the information in the memory cell. In addition, utilizing the LSTM module increases the network depth while the number of the required parameters remains low. Having a deeper network enables extracting more complex features and this is the main objective of our models as the negative set contains hard samples.

The Keras framework is used for constructing and training the proposed models (Chollet F. et al., 2015). Adam optimizer (Kingma and Ba, 2014) is used for updating the parameters with a learning rate of 0.001. The batch size is set to 32 and the number of epochs is set to 50. Early stopping is applied based on validation loss.

## 3. Results and Discussion

### 3.1. Performance Measures

In this work, we use the widely adopted evaluation metrics for evaluating the performance of the proposed models. These metrics are precision, recall, and Matthew correlation coefficient (MCC), and they are defined as follows:

$$\begin{array}{r} Precision=\frac{TP}{TP+FP} \end{array}$$

$$\begin{array}{r} Recall=\frac{TP}{TP+FN} \end{array}$$

$$\begin{array}{r} Mcc=\frac{TP\times TN-FP\times FN}{\sqrt{\left(TP+FP\right)\left(TP+FN\right)\left(TN+FP\right)\left(TN+FN\right)}}\; \end{array}$$

Where TP is true positive and represents correctly identified promoter sequences, TN is true negative and represents correctly rejected promoter sequences, FP is false positive and represents incorrectly identified promoter sequences, and FN is false negative and represents incorrectly rejected promoter sequences.

### 3.2. Effect of the Negative Set

When analyzing the previously published works for promoter sequences identification we noticed that the performance of those works greatly depends on the way of preparing the negative dataset. They performed very well on the datasets that they have prepared, however, they have a high false positive ratio when evaluated on a more challenging dataset that includes non-prompter sequences having common motifs with promoter sequences. For instance, in case of the TATA promoter dataset, the randomly generated sequences will not have TATA motif at the position -30 and –25 bp which in turn makes the task of classification easier. In other words, their classifier depended on the presence of TATA motif to identify the promoter sequence and as a result, it was easy to achieve high performance on the datasets they have prepared. However, their models failed dramatically when dealing with negative sequences that contained TATA motif (hard examples). The precision dropped as the false positive rate increased. Simply, they classified these sequences as positive promoter sequences. A similar analysis is valid for the other promoter motifs. Therefore, the main purpose of our work is not only achieving high performance on a specific dataset but also enhancing the model ability on generalizing well by training on a challenging dataset.

**Figure 5:**
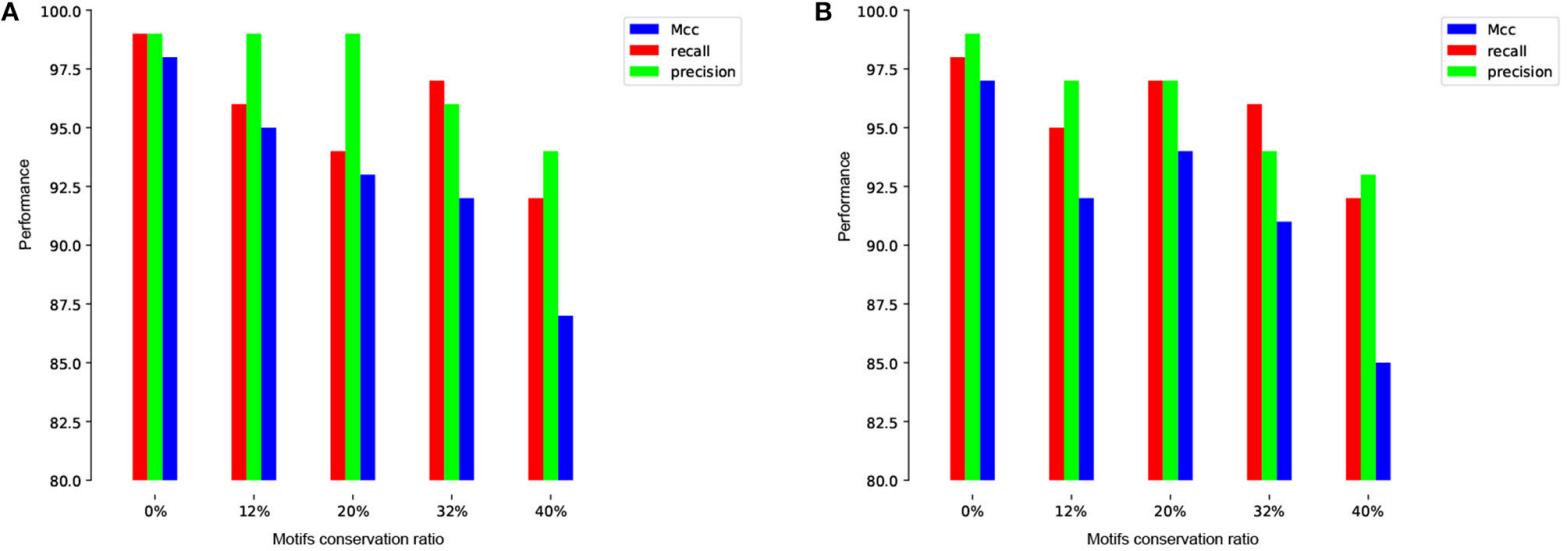
The effect of different conservation ratios of TATA motif in the negative set on the performance in case of TATA promoter dataset for both human **(A)** and mouse **(B)**.

To more illustrate this point, we train and test our model on the human and mouse TATA promoter datasets with different methods of negative sets preparation. The first experiment is performed using randomly sampled negative sequences from non-coding regions of the genome (i.e., similar to the approach used in the previous works). Remarkably, our proposed model achieves nearly perfect prediction accuracy (precision=99%, recall=99%, Mcc=98%) and (precision=99%, recall=98%, Mcc=97%) for both human and mouse, respectively. These high results are expected, but the question is whether this model can maintain the same performance when evaluated on a dataset that has hard examples. The answer, based on analyzing the prior models, is no. The second experiment is performed using our proposed method for preparing the dataset as explained in section 2.2. We prepare the negative sets that contain conserved TATA-box with different percentages such as 12, 20, 32, and 40% and the goal is reducing the gap between the precision and the recall. This ensures that our model learns more complex features rather than learning only the presence or absence of TATA-box. As shown in Figures 5A,B the model stabilizes at the ratio 32~40% for both human and mouse TATA promoter datasets.

### 3.3. Results and Comparison

Over the past years, plenty of promoter region prediction tools have been proposed (Hutchinson, 1996; Scherf et al., 2000; Reese, 2001; Umarov and Solovyev, 2017). However, some of these tools are not publically available for testing and some of them require more information besides the raw genomic sequences. In this study, we compare the performance of our proposed models with the current state-of-the-art work, CNNProm, which was proposed by Umarov and Solovyev (2017) as shown in Table 2. Generally, the proposed models, DeePromoter, clearly outperform CNNProm in all datasets with all evaluation metrics. More specifically, DeePromoter improves the precision, recall, and MCC in the case of human TATA dataset by 0.18, 0.04, and 0.26, respectively. In the case of human non-TATA dataset, DeePromoter improves the precision by 0.39, the recall by 0.12, and MCC by 0.66. Similarly, DeePromoter improves the precision, and MCC in the case of mouse TATA dataset by 0.24 and 0.31, respectively. In the case of mouse non-TATA dataset, DeePromoter improves the precision by 0.37, the recall by 0.04, and MCC by 0.65. These results confirm that CNNProm fails to reject negative sequences with TATA promoter, therefore, it has high false positive. On the other hand, our models are able to deal with these cases more successfully and false positive rate is lower compared with CNNProm.

**Table 2:** Comparison of the DeePromoter with the state-of-the-art method.

**Oganism**	**Method**	**Precision**	**Recall**	**Mcc**
	DeePromoter	**0.93**	**0.95**	**0.88**
Human TATA	CNNProm	0.75	0.91	0.62
	DeePromoter	**0.97**	**0.95**	**0.92**
Human non-TATA	CNNProm	0.58	0.83	0.26
	DeePromoter	**0.92**	0.95	**0.87**
Mouse TATA	CNNProm	0.68	**0.96**	0.56
	DeePromoter	**0.91**	**0.90**	**0.82**
Mouse non-TATA	CNNProm	0.54	0.86	0.17

**Figure 6:**
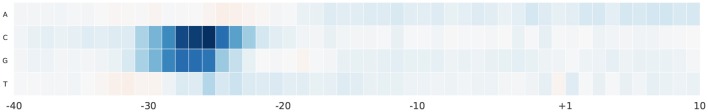
The saliency map of the region –40 bp to 10 bp, which includes the TATA-box, in case of human TATA promoter sequences.

**Figure 7:**
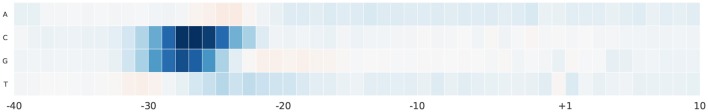
The saliency map of the region –40 bp to 10 bp, which includes the TATA-box, in case of mouse TATA promoter sequences.

For further analyses, we study the effect of alternating nucleotides at each position on the output score. We focus on the region –40 and 10 bp as it hosts the most important part of the promoter sequence. For each promoter sequence in the test set, we perform computational mutation scanning to evaluate the effect of mutating every base of the input subsequence (150 substitutions on the interval –40~10 bp subsequence). This is illustrated in Figures 6, 7 for human and mouse TATA datasets, respectively. Blue color represents a drop in the output score due to mutation while the red color represents the increment of the score due to mutation. We notice that altering the nucleotides to C or G in the region –30 and –25 bp reduces the output score significantly. This region is TATA-box which is a very important functional motif in the promoter sequence. Thus, our model is successfully able to find the importance of this region. In the rest of the positions, C and G nucleotides are more preferable than A and T, especially in case of the mouse. This can be explained by the fact that the promoter region has more C and G nucleotides than A and T (Shi and Zhou, 2006).

## 4. Conclusion

Accurate prediction of promoter sequences is essential for understanding the underlying mechanism of the gene regulation process. In this work, we developed DeePromoter -which is based on a combination of convolution neural network and bidirectional LSTM- to predict the short eukaryote promoter sequences in case of human and mouse for both TATA and non-TATA promoter. The essential component of this work was to overcome the issue of low precision (high false positive rate) noticed in the previously developed tools due to the reliance on some obvious feature/motifs in the sequence when classifying promoter and non-promoter sequences. In this work, we were particularly interested in constructing a hard negative set that drives the models toward exploring the sequence for deep and relevant features instead of only distinguishing the promoter and non-promoter sequences based on the existence of some functional motifs. The main benefits of using DeePromoter is that it significantly reduces the number of false positive predictions while achieving high accuracy on challenging datasets. DeePromoter outperformed the previous method not only in the performance but also in overcoming the issue of high false positive predictions. It is projected that this framework might be helpful in drug-related applications and academia.

## Author Contributions

MO and ZL prepared the dataset, conceived the algorithm, and carried out the experiment and analysis. MO and HT prepared the webserver and wrote the manuscript with support from ZL and KC. All authors discussed the results and contributed to the final manuscript.

### Conflict of Interest Statement

The authors declare that the research was conducted in the absence of any commercial or financial relationships that could be construed as a potential conflict of interest.
